# Diffusion-weighted MRI radiomics of spine bone tumors: feature stability and machine learning-based classification performance

**DOI:** 10.1007/s11547-022-01468-7

**Published:** 2022-03-23

**Authors:** Salvatore Gitto, Marco Bologna, Valentina D. A. Corino, Ilaria Emili, Domenico Albano, Carmelo Messina, Elisabetta Armiraglio, Antonina Parafioriti, Alessandro Luzzati, Luca Mainardi, Luca Maria Sconfienza

**Affiliations:** 1 Dipartimento Di Scienze Biomediche Per La Salute, Università Degli Studi Di Milano, Via Riccardo Galeazzi 4, 20161 Milan, Italy; 2 Department of Electronics, Information and Bioengineering (DEIB), Politecnico Di Milano, Via Golgi 39, 20133 Milan, Italy; 3 Scuola Di Specializzazione in Radiodiagnostica, Università Degli Studi Di Milano, 20122 Milan, Italy; 4 Sezione Di Scienze Radiologiche, Dipartimento Di Biomedicina, Neuroscienze E Diagnostica Avanzata, Università Degli Studi Di Palermo, 90127 Palermo, Italy; 5 IRCCS Istituto Ortopedico Galeazzi, 20161 Milan, Italy; 6 Pathology Department, ASST Pini-CTO, 20122 Milan, Italy

**Keywords:** Machine learning, Radiomics, Reproducibility, Spine, Texture analysis, Tumor

## Abstract

**Purpose:**

To evaluate stability and machine learning-based classification performance of radiomic features of spine bone tumors using diffusion- and T2-weighted magnetic resonance imaging (MRI).

**Material and methods:**

This retrospective study included 101 patients with histology-proven spine bone tumor (22 benign; 38 primary malignant; 41 metastatic). All tumor volumes were manually segmented on morphologic T2-weighted sequences. The same region of interest (ROI) was used to perform radiomic analysis on ADC map. A total of 1702 radiomic features was considered. Feature stability was assessed through small geometrical transformations of the ROIs mimicking multiple manual delineations. Intraclass correlation coefficient (ICC) quantified feature stability. Feature selection consisted of stability-based (ICC > 0.75) and significance-based selections (ranking features by decreasing Mann–Whitney p-value). Class balancing was performed to oversample the minority (i.e., benign) class. Selected features were used to train and test a support vector machine (SVM) to discriminate benign from malignant spine tumors using tenfold cross-validation.

**Results:**

A total of 76.4% radiomic features were stable. The quality metrics for the SVM were evaluated as a function of the number of selected features. The radiomic model with the best performance and the lowest number of features for classifying tumor types included 8 features. The metrics were 78% sensitivity, 68% specificity, 76% accuracy and AUC 0.78.

**Conclusion:**

SVM classifiers based on radiomic features extracted from T2- and diffusion-weighted imaging with ADC map are promising for classification of spine bone tumors. Radiomic features of spine bone tumors show good reproducibility rates.

**Supplementary Information:**

The online version contains supplementary material available at 10.1007/s11547-022-01468-7.

## Introduction

Bone tumors of the spine include benign and malignant entities. The incidence of benign lesions, such as hemangioma, is hard to determine as they are often asymptomatic and remain undetected or are discovered incidentally. Among malignant bone tumors of the spine, metastatic lesions are far more common than primary lesions [1]. Imaging, and particularly magnetic resonance imaging (MRI), plays a pivotal role in the discrimination among these entities [2]. Although MRI performed with conventional pulse sequences has good reliability, some features of benign and malignant spinal lesions overlap and make the differential diagnosis challenging [3]. Diffusion-weighted (DW) imaging provides information regarding tumor cellularity. Specifically, apparent diffusion coefficient (ADC) maps are quantitative measures of the magnitude of diffusion within tissues. The role of DW imaging has been highlighted in previous studies dealing with bone tumors of the spine [4], and mean ADC values have been shown to discriminate between benign and malignant entities, including both primary malignant and metastatic lesions [5].

Radiomics includes extraction, analysis and interpretation of large numbers of quantitative data, known as radiomic features, from medical imaging [6, 7]. Radiomics has gained attention in oncologic imaging, and most studies to date have focused on discriminating tumor grades and types before treatment, monitoring response to therapy and predicting outcome [8]. A great variability in radiomic features has, however, emerged as a major issue across these studies, particularly with regard to the segmentation process, thus highlighting the need for preliminary assessment of feature stability [9]. Machine learning aids in analyzing radiomic data, as it performs inferences from large amounts of radiomic features and creates classification models for the diagnosis of interest [10, 11].

The objectives of this study were to evaluate stability and machine learning-based classification performance of radiomic features of spine bone tumors extracted from DW and T2-weighted magnetic resonance imaging (MRI). ADC value differences among benign, primary malignant and metastatic tumors of the spine were also assessed.

## Materials and methods

### Dataset description

Institutional Review Board approval and a waiver for informed consent were obtained. This retrospective study included 101 patients with histology-proven spine bone tumors and DW MRI performed over the last 10 years at one tertiary bone tumor center. Of the 101 patients used for the study, 22 were benign and 79 were malignant (38 primary and 41 metastatic). Clinical and demographic characteristics are summarized in Table 1.

**Table 1:** Main demographic and clinical data involved in the project

Demographic and Clinical Data			
Age (median [IQR])		58 years [46–67]	
Sex		49 Male; 52 Female	
Tumor type	Benign (n = 22)	Primary malignant (n = 38)	Bone metastases (n = 41)
	Fibrous dysplasia (n = 3) Giant cell tumors (n = 2) Hemangioma (n = 7) Osteoblastoma (n = 10)	Chondrosarcoma (n = 3) Chordoma (n = 10) Ewing sarcoma (n = 5) Lymphoma (n = 8) Multiple myeloma (n = 12)	Breast (n = 14) Kidney (n = 6) Lung (n = 13) Thyroid (n = 3) Gastrointestinal tract (n = 5)

IQR: interquartile range

All images were acquired using 1.5 T MRI scanners and different image acquisition parameters (pixel spacing, slice thickness, time of repetition and echo). T2-weighted and DW images were available for all patients included. T2-weighted images were acquired using turbo spin-echo pulse sequences, while DW images were acquired using echo-planar imaging with b-values of 0 and 1000 s/mm^2^. Further details on MRI acquisition are displayed in Table 2. The DW images were used to fit ADC maps.

**Table 2:** Acquisition information for the magnetic resonance images used for this study. For the numerical variables, the full range of values was represented

MRI Acquisition Info		
Image type	T2w	DWI/ADC
Scanner	- Siemens Avanto: 80 patients - Siemens Espree: 18 patients - Philips Ingenia: 2 patients - Philips Achieva: 1 patient	
Magnetic field	1.5 T	
Pulse sequence	Turbo spin-echo	Echo-planar imaging
Echo train length	13–59	1
Number of averaging	1–10	2–6
Time of repetition (ms)	2000–10,360	2700–10,700
Time of echo (ms)	69–117	65–94
Slice thickness (mm)	2–5	3–6
Pixel spacing (mm)	0.36–1.45	1.30–2.57
Slice spacing (mm)	2.2–7	3.3–7.8
Flip Angle (°)	90–150	90

T2w: T2-weighted; DWI: diffusion-weighted images; ADC: apparent diffusion coefficient maps

### Image segmentation

**Fig. 1:**
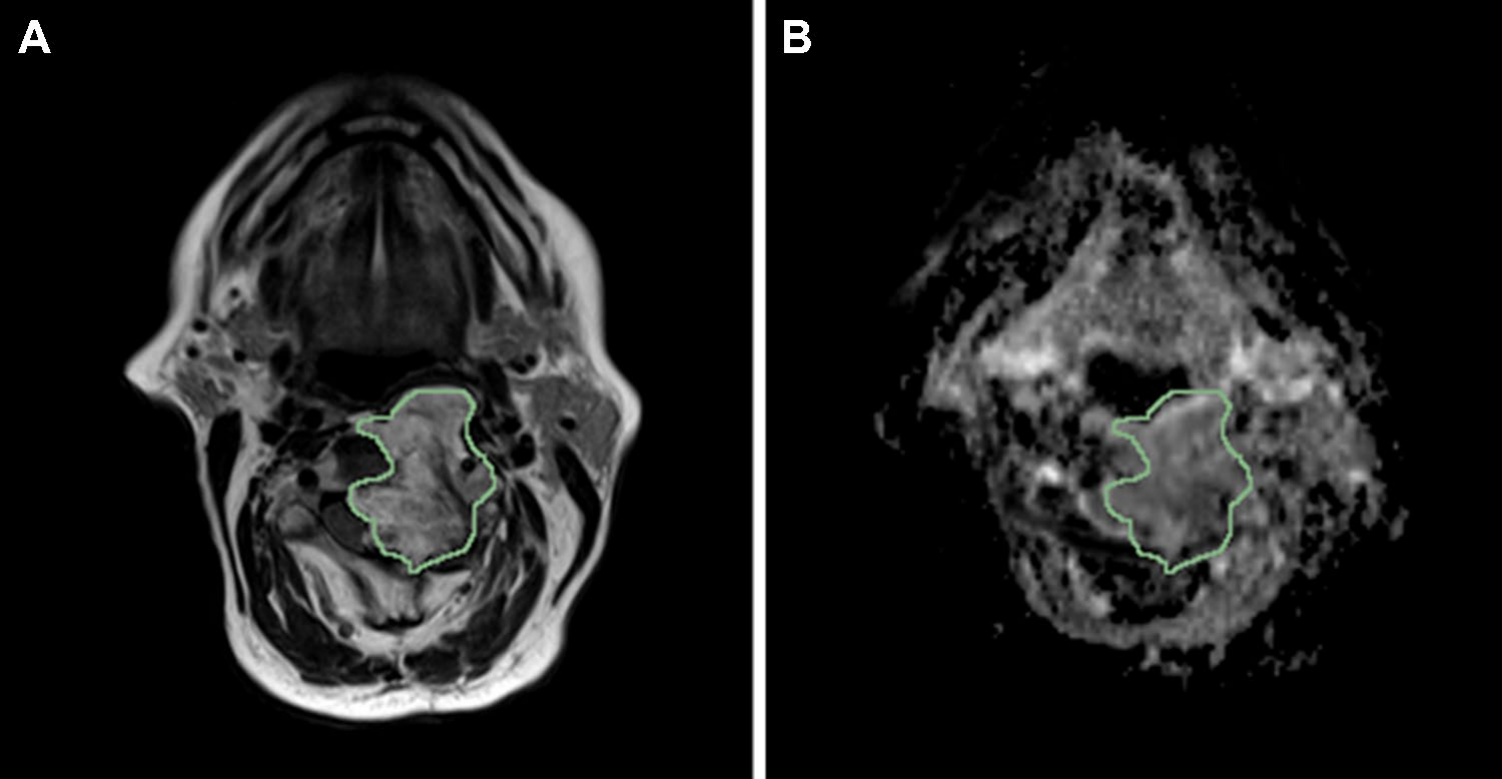
Example of tumor segmented by the radiologist. The tumor is segmented on the T2-weighted image **(a)** but the same segmentation has been used as a mask to extract the radiomic features from the apparent diffusion coefficient map **(b)** as well

3D image segmentation was manually performed by a last-year resident in radiology experienced in musculoskeletal and oncologic imaging using the open-source software ITK-SNAP (version 3.6) [12]. The reader drew a region of interest (ROI) along tumor borders on each slice using axial T2-weighted sequence, which provided more morphological details compared to DW images. Since there was not an excessive movement of the patient from one sequence to the other, the same segmentation was used as a mask to extract the radiomic features from both ADC and T2-weighted images. An example of segmentation overlaid on both T2-weighted and ADC images is displayed in Fig. 1.

### Image preprocessing

Different preprocessing steps were applied to the MRI images prior to the extraction of the radiomic features. First, a 3D Gaussian filter with a 3 × 3× 3 voxel kernel and σ = 0.5 was used to denoise the images. In case of T2-weighted images, a bias field correction was performed using the N4ITK algorithm [13] to correct for potential effects due to inhomogeneity of the magnetic field. Also, Z-score standardization was performed to ensure that the range of intensity in the T2-weighted images was the same. Last, the images were resampled to a common isotropic resolution of 2 mm (as in [14]) using B-spline interpolation. Bias field correction and intensity standardization were not applied on the ADC maps because those types of images already have a standardized range of intensity (diffusion coefficients are within the maximum range of 0–4 10^–3^ mm^2^/s) and are not affected magnetic field inhomogeneity.

### Radiomic features extraction

An initial set of 1702 radiomic features was extracted from the MRI volumes (851 features per sequence) using the PyRadiomics library (version 3.0) [15]. Features belonged to different categories such as shape and size, first-order statistics (FOS) and textural features. Textural features were computed using five different textural matrices: the gray-level co-occurrence matrix (GLCM); the gray-level run length matrix (GLRLM); the gray-level size zone matrix (GLSZM); the neighboring gray tone difference matrix (NGTDM); and the gray-level dependence matrix (GLDM). FOS and textural features were extracted from both the original volumes and the 8 volumes obtained by first-level wavelet decomposition [16]. For the full list of radiomic features, refer to PyRadiomics documentation (https://pyradiomics.readthedocs.io/en/latest/features.html) and to supplementary material “Supplementary 1 – Features description.” A 32-bins gray-level discretization was used prior to the radiomic features extraction.

### Stability analysis

**Fig. 2:**
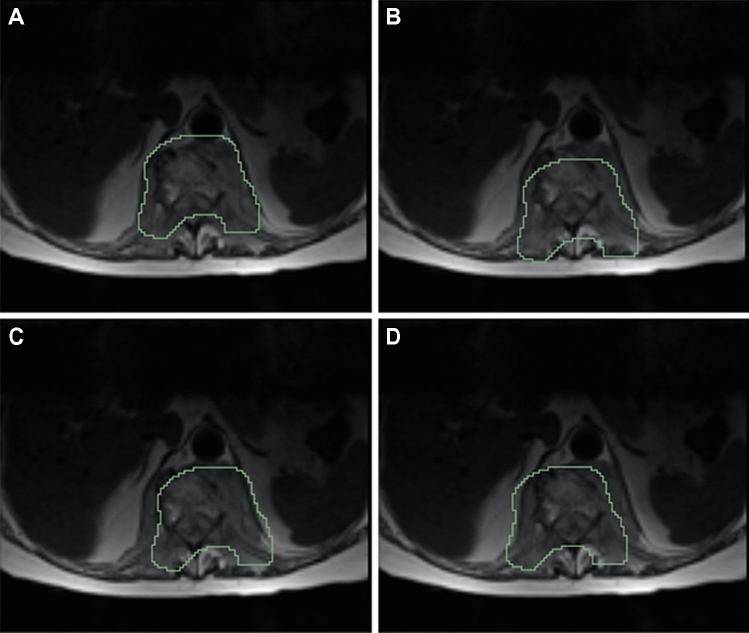
Translated versions of the same region of interest used for the stability analysis. **(a)** Upward translation. **(b)** Downward translation. **(c)** Translation to the right. **(d)** Translation to the left

Prior to the training of any radiomic model, a side experiment was performed to evaluate the stability of radiomic features to variations in the ROI. This stability analysis was performed to mimic the effect of potential sources of variability such as the intra- and inter-reader variability that comes with manual segmentation or the potential mismatch between the ROI and the underlying tumor when the same ROI is used to extract features from multiple sequences; like it was done in this study. The stability analysis was performed as described in [17], by applying different translation of the ROI in the positive and negative direction of the x and y directions, respectively (Fig. 2). The entity of the translation was 10% of the length of the bounding box of the tumor in the corresponding direction (x or y). For each patient, the radiomic features were extracted from 5 different ROIs (the original and the 4 translated versions). Intraclass correlation coefficient (ICC) was used to quantify the stability of each radiomic feature [18]. A feature was considered stable if ICC was higher than 0.75 [19].

### Radiomic classifier training and validation pipeline

**Fig. 3:**
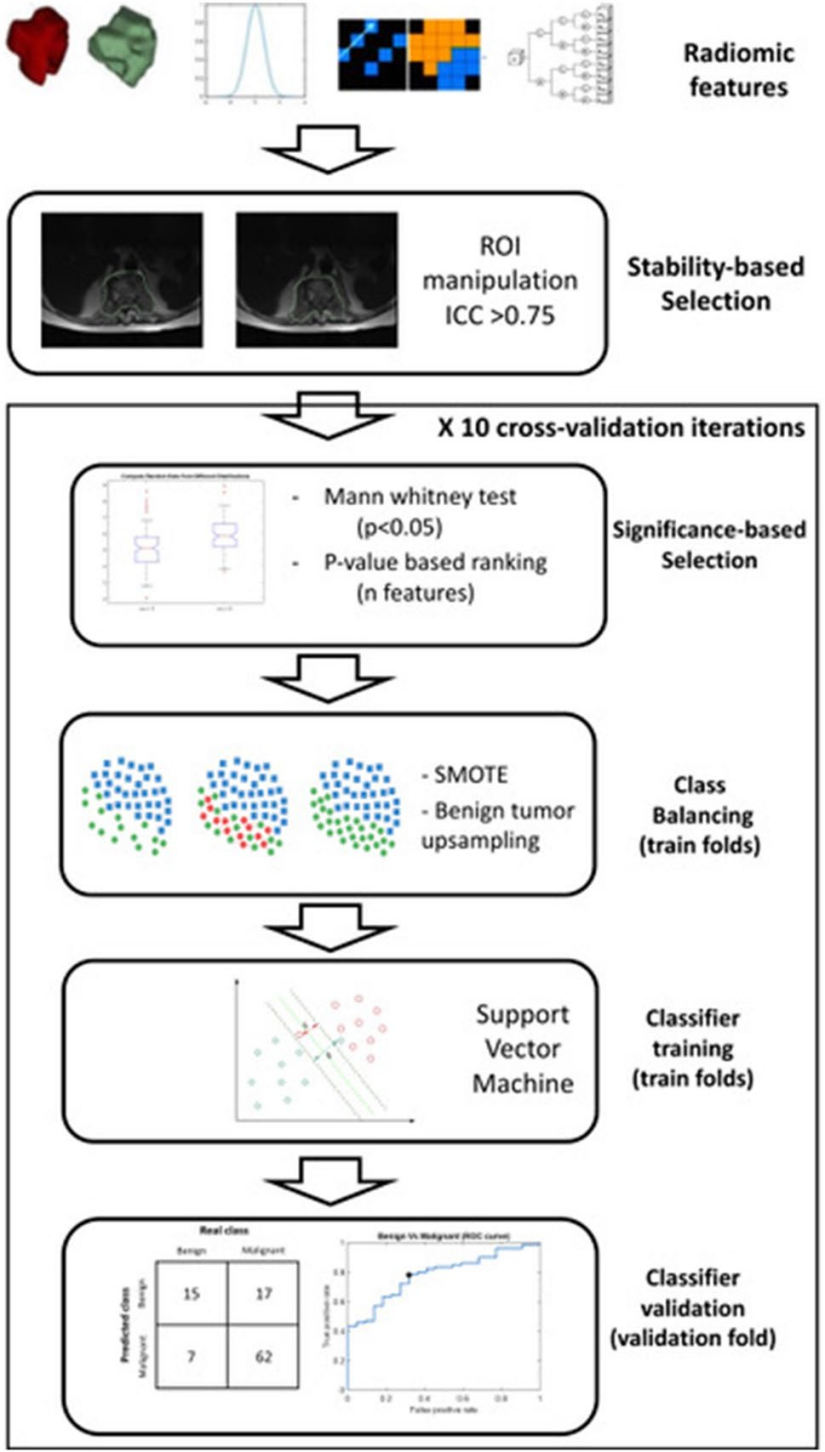
Workflow of the radiomic classifier training

Figure 3 shows the scheme including all the steps for the training and validation of the radiomics-based classifier. First, according to the previous stability analysis, radiomic features having an ICC of 0.75 or lower were consider unstable and excluded. The second step of features selection was related to the ability of radiomic features to discriminate between malignant and benign tumors. Mann–Whitney tests were used to identify features with a statistically different distribution between the two groups. The statistically different features were kept and ranked by their p-value (from lower to higher). Of these ranked features, only the first *n* were selected (with *n* ranging from 1 to 10).

Prior to the training of the classifier, a class balancing was applied using the Synthetic Minority Oversampling Technique (SMOTE) [20], which is a technique used to artificially oversample the minority class (in this case, the benign tumors). The balanced dataset was used to train a support vector machine (SVM) classifier to discriminate the type of tumor. The reference “positive” group for the training of the classifier was the group of malignant tumors. The training was performed in MATLAB using *fitcsvm* function with the default hyperparameters (see Name-Value Pair Arguments in https://it.mathworks.com/help/stats/fitcsvm.html) and a linear kernel.

### Radiomic classifier validation

The diagnostic performance of the radiomic model was evaluated using tenfold cross-validation on the training set. In each iteration, the training pipeline was applied to 9/10 of the dataset and the train model was used to classify the remaining patients. For each patient, an unbiasedly evaluated class and a classification score could be computed. Using these two elements, both a confusion matrix and a receiver operating characteristics (ROC) curve could be computed. Sensitivity, specificity and accuracy of the classification as well as the area under the ROC curve (AUC) were determined and used to evaluate the quality of the classifier. In the computation of these metrics, the group of malignant tumors was considered as the “positive” class. The number of selected features used to train the different model was varied from 1 to 10 to evaluate the effect of an increasing number of features on model’s performance.

### Discriminative power of mean ADC

Since previous studies [5] reported the ability of mean ADC in discriminating the different categories of tumor (benign, primary malignant or metastatic), we tried to confirm this ability, using the mean ADC obtained by the ROI of this dataset. A Kruskal–Wallis test and post hoc comparisons were performed to evaluate whether mean ADC had a significantly different distribution among the groups. Also, the AUC of mean ADC for the benign vs. malignant classification was evaluated.

## Results

### Stability analysis

A total of 1300 extracted radiomic features (76.4%) were stable to transformations of the ROI. The full list of radiomic features used for the following analysis is detailed in supplementary material “Supplementary 2—Features stability.xlsx.”

### Radiomic classifier validation

**Fig. 4:**
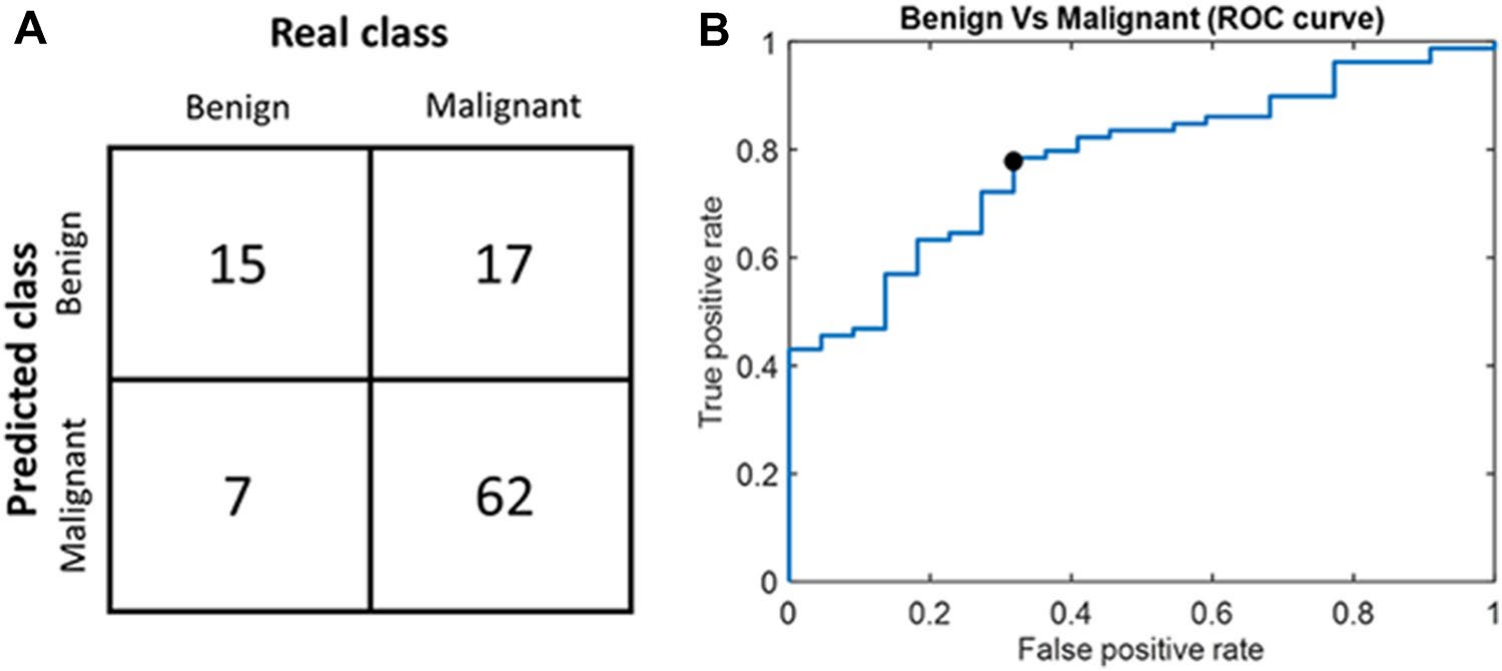
Results of the best radiomic classifiers. **(a)** Confusion matrix on which sensitivity, specificity and accuracy have been computed. **(b)** Receiver operating characteristic (ROC) curve, with the black dot representing the actual sensitivity and specificity of the classifier

The quality metrics for the SVM models are displayed in Table 3 as a function of the number of selected features. The model with the best performance and the lowest number of features was the model with 8 features, with a sensitivity of 0.78, specificity of 0.68 and accuracy of 0.76 (Fig. 4a). The ROC curve of the best model is displayed in Fig. 4b. Its area under the ROC curve (AUC) was 0.78.

**Table 3:** Results of the different classifiers tested as a function of the number of features

Model Performance by Number of Features				
Features number	Sensitivity	Specificity	Accuracy	AUC
1	0.80	0.59	0.75	0.77
2	0.80	0.59	0.75	0.78
3	0.78	0.64	0.75	0.78
4	0.77	0.55	0.72	0.77
5	0.76	0.59	0.72	0.78
6	0.76	0.59	0.72	0.78
7	0.77	0.64	0.74	0.78
8	**0.78**	**0.68**	**0.76**	**0.78**
9	0.77	0.59	0.73	0.78
10	0.78	0.59	0.74	0.77

The best model is highlighted in bold

AUC: area under the ROC curve

### Discriminative power of mean ADC

**Fig. 5:**
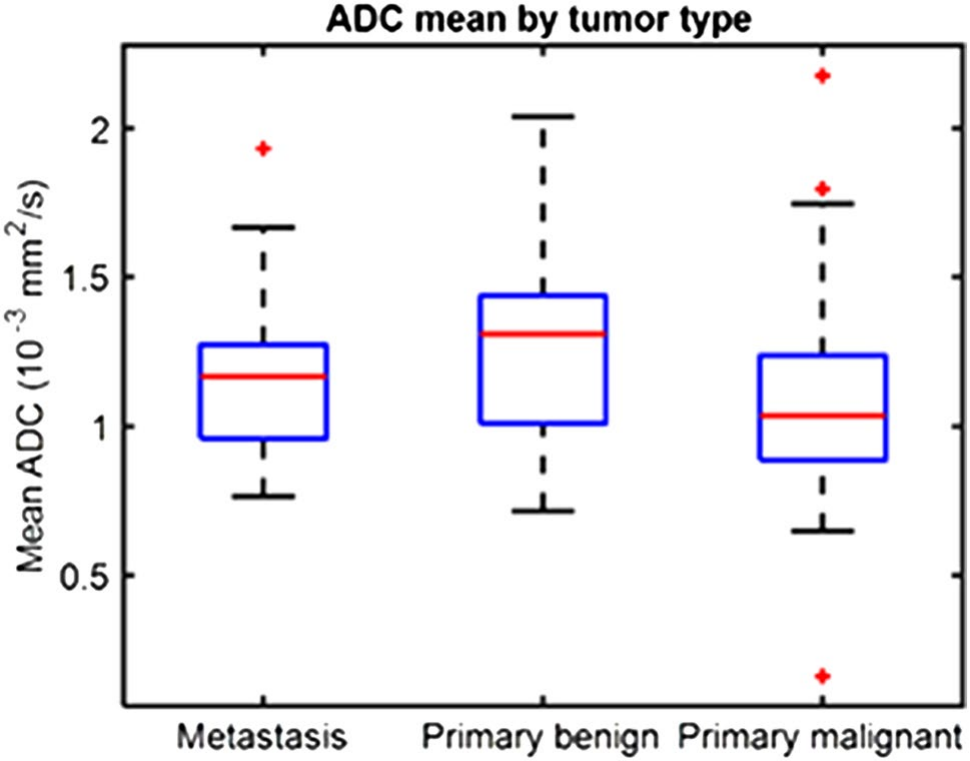
Boxplots showing the distribution of the values of mean apparent diffusion coefficient (ADC). **(a)** Values of this study. **(b)** Distributions observed in [5]

The boxplots in Fig. 5a show the distribution of mean ADC among the different groups of tumors (benign, primary malignant and metastasis). The values of mean ADC were 1.30 ± 0.35 *10^–3^ mm^2^/s for benign tumors, 1.17 ± 0.24 *10^–3^ mm^2^/s for metastasis and 1.08 ± 0.36 *10^–3^ mm^2^/s for primary malignant tumors. The p-value for the Kruskal–Wallis test was 0.02, and post hoc comparisons showed that the difference between benign and primary malignant tumors was statistically significant (p = 0.01) but not between benign tumors and metastasis (*p* = 0.38). The AUC of mean ADC for benign vs. malignant tumor was 0.66.

## Discussion

The main finding of this study is that SVM models based on radiomic features derived from T2-weighted and DW MRI with ADC map allowed for benign vs. malignant classification of spine bone tumors with up to 76% accuracy. Additionally, the reproducibility rate of radiomic features was higher than 76% as features were stable to geometrical transformations of the ROIs.

Previous studies dealt with MRI radiomics-based classification of spine bone tumors. In cancer patients with bone marrow metastatic disease, a very preliminary investigation showed that MRI-based radiomic signature could be helpful to discriminate between metastatic and non-metastatic vertebral bodies [21]. Recently, Chianca et al. tested different extraction software for radiomics-based classification of spine bone tumors using T1-weighted and T2-weighted MRI and reported that PyRadiomics outperformed other extraction software [22]. The reason is likely that PyRadiomics directly enables robust image preprocessing, thus removing dependencies on some image parameters and allowing for generalizability of the results, as it was done in our study. Lang et al. [23] focused on dynamic contrast-enhanced MRI and differentiated spine metastatic lesions originated from lung and other cancers using radiomics and deep learning. In a dataset of 30 metastases from lung cancer and 31 metastases from other cancers, classification using convolutional neural networks achieved a mean accuracy of 71% [23]. Our current study adds to the literature by highlighting the role of machine-learning classification of spine bone tumors based on radiomic features extracted from DW MRI with ADC maps, coupled with morphologic T2-weighted sequences. A SVM was trained and tested based on selected radiomic features and had an accuracy of up to 76%.

Stability analysis allows for assessing the robustness of radiomic features and represents a preliminary step in the process of feature selection. Several strategies can be used for stability evaluation, such as changes in image acquisition parameters [24] and multiple ROI delineations performed by different readers [25], which are, however, time-consuming. In our study, feature stability was assessed through a time-saving method based on geometrical transformations of the ROIs mimicking multiple manual delineations [17]. More than 76% radiomic features were stable to these transformations and then showed good overall reproducibility.

A recent meta-analysis showed that quantitative assessment of ADC was excellent for differentiating benign from malignant bone marrow lesions of the spine [4]. In a study included in this meta-analysis, mean ADC values were found to be higher in benign bone tumors in comparison with both primary malignant and metastatic lesions of the spine [5]. Our results are in line with those findings (Fig. 5B), as benign tumors showed higher ADC values than primary malignant lesions and metastases, although statistical significance was not reached in the latter case. The AUC of mean ADC for benign vs. malignant tumor discrimination was 0.66 and 0.76 in our and previous [5] studies, respectively. However, an overlap between mean ADC values of benign and malignant tumors was highlighted in the previous investigation, e.g., giant cell tumor of the bone is histologically benign but has low ADC mean value [5]. In this regard, an objective assessment of tumor heterogeneity by means of radiomics and machine learning might aid in the diagnosis.

Some limitations of this study need to be taken into account. First, it has a retrospective design as a prospective analysis is not strictly necessary for radiomic studies [8]. Second, the number of histology-proven benign lesions was small, i.e., approximately one fifth of the population of study, because histology was not available for unbiopsied benign lesions with typical imaging findings. However, this limitation was overcome by means of class balancing with SMOTE technique [20]. Finally, an independent cohort of patients was not available for external validation of the radiomics-based classifier and needs to be included in further investigations.

Limitations notwithstanding, we conclude that SVM models based on radiomic features extracted from T2-weighted and DW MRI with ADC map are promising for classification of bone tumors of the spine and radiomic features show good overall reproducibility.

## Supplementary Information

Below is the link to the electronic supplementary material.

Supplementary file1 (DOCX 1161 kb)

Supplementary file2 (XLSX 47 kb)

## Data Availability

Data may be obtained from the corresponding author on reasonable request.

## Abbreviations

ADC: Apparent diffusion coefficient
AUC: Area under the ROC curve
DW: Diffusion weighted
FOS: First-order statistics
GLCM: Gray-level co-occurrence matrix
GLDM: Gray-level dependence matrix
GLRLM: Gray-level run length matrix
GLSZM: Gray-level size zone matrix
ICC: Intraclass correlation coefficient
MRI: Magnetic resonance imaging
NGTDM: Neighboring gray tone difference matrix
ROC: Receiver operating characteristics
ROI: Region of interest
SMOTE: Synthetic minority oversampling technique
SVM: Support vector machine

## Code availability

Data may be obtained from the corresponding author on reasonable request.
