# Supplementary material for: Diffusion-weighted MRI radiomics of spine bone tumors: feature stability and machine learning-based classification performance
Source: Radiol Med. 2022 Mar 23;127(5):518–25. doi: 10.1007/s11547-022-01468-7 (PMC9098537; doi:10.1007/s11547-022-01468-7)
Supplement: Supplementary file 1 — Supplementary file1 (DOCX 1161 kb) [file 11547_2022_1468_MOESM1_ESM.docx]

**Radiomic features description**

The purpose of this section is to provide a general knowledge of the different mathematical tools that are used to compute the radiomic features used within the study but not to provide the complete list of all the radiomic features. For that, the reader may refer to the documentation of Pyradiomics [1], or to the manual of the Imaging Biomarker Standardization Initiative (IBSI, see [2]).

***Shape and size features***

Shape and Size features (SS) describe geometric aspects of a region of interest (ROI), such as area and volume. To calculate these features, different representation of the ROI may be used [2]:

- A collection of voxels with each voxel taking up a certain volume.
- A voxel point set that consists of coordinates of the voxel centers.
- A surface mesh.

SS features in Pyradiomics are typically computed using a surface mesh. A surface mesh is a surface made of M adjacent triangular surfaces and N vertices. Each mesh can be described using a Nx3 matrix with the 3D coordinates of the points and a Mx3 matrix with the index of the points

that are used to define each surface. SS features may be computed both in 3D and 2D but for this thesis, only 3D features were used.

***First order statistics***

First Order Statistics (FOS) are features that describe the distribution of the different grey values inside the ROI. Features of this category may be further classified in intensity-based or histogram-based features [2]. The difference between the two is that to compute the latter, a histogram is

required. The computation of the histogram requires the discretization of the distribution of the grey levels in bins. Two approaches can be used for the histogram discretization [2]: fixed bin size and fixed bin number.

Intensity-based statistical features are not meaningful if the intensity scale is arbitrary, like in MRI. So proper intensity standardization should be made before any radiomic analysis.

***Textural features and textural matrices***

Textural features provide spatial information about the distribution of the grey values inside the ROI [67]. Just as some FOS features are computed from a histogram, textural features are computed from textural matrices. Like for histogram-based features, grey values discretization is usually performed prior to the computation of the textural matrices.

In this study, 5 different textural matrices were used: Grey Level Cooccurrence Matrix (GLCM); Grey Level Run Length Matrix (GLRLM); Grey Level Size Zone Matrix (GLSZM); Neighboring Grey Tone Difference Matrix (NGTDM); Grey Level Dependence Matrix (GLDM).

The GLCM is a N_g_-by-N_g_ matrix, N_g_ being the number of discrete grey values in an image and describes the second order joint probability function of an image region constrained by a ROI. Fixed a given positive distance δ and a direction θ the (i; j) element of the GLCM describes how many times the element grey value j appears at a distance δ (e.g. one pixel) from the grey value i in the direction θ (e.g. horizontal). An example of GLCM computation is reported in Figure S1. The rules for GLCM computation are analogous in 3D, with the only difference in the number of possible θ directions (13 instead of 4).

The GLRLM is a matrix that quantifies grey level runs, which are defined as the length of consecutive pixels that have the same grey level value. Given a direction θ, the (i; j) element of the GLRLM describes how many times the element grey value i appears consecutively for j times. Therefore,

the dimension of the matrix is N_g_-by-N_max_, N_max_ being the maximum size in the image. An example of GLRLM computation is reported in Figure S2.


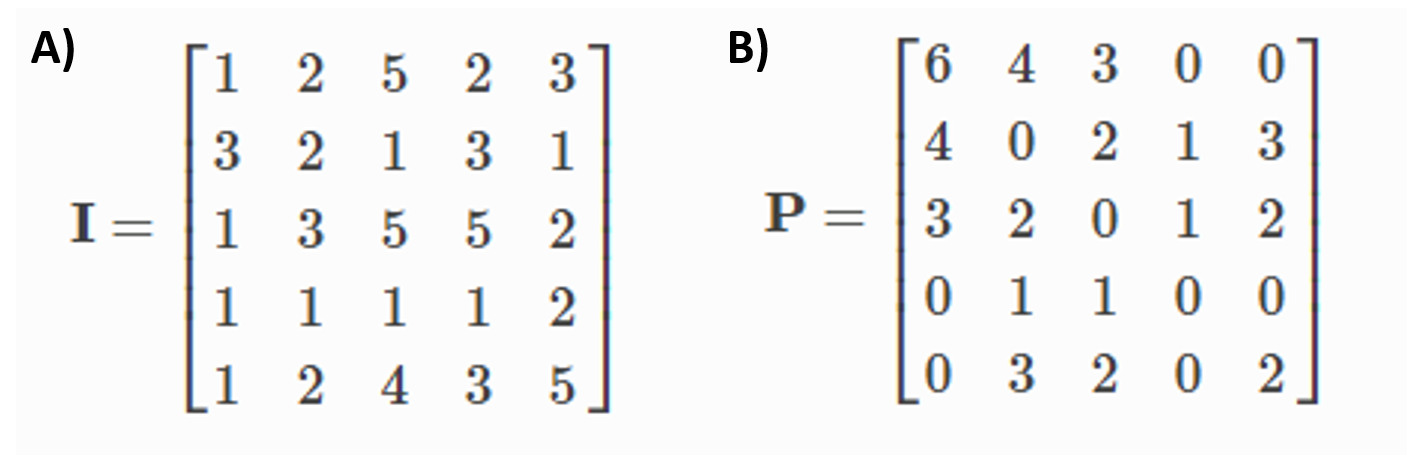


**Figure S1** Example of computation of a Grey Level Co-occurrence Matrix (GLCM). A) Image I with the original grey values. B) Corresponding GLCM. The matrix P in B) is computed using δ = 1 (1-pixel distance) and θ = 0 (horizontal direction, both left to right and right to left). Adapted from [1].

*
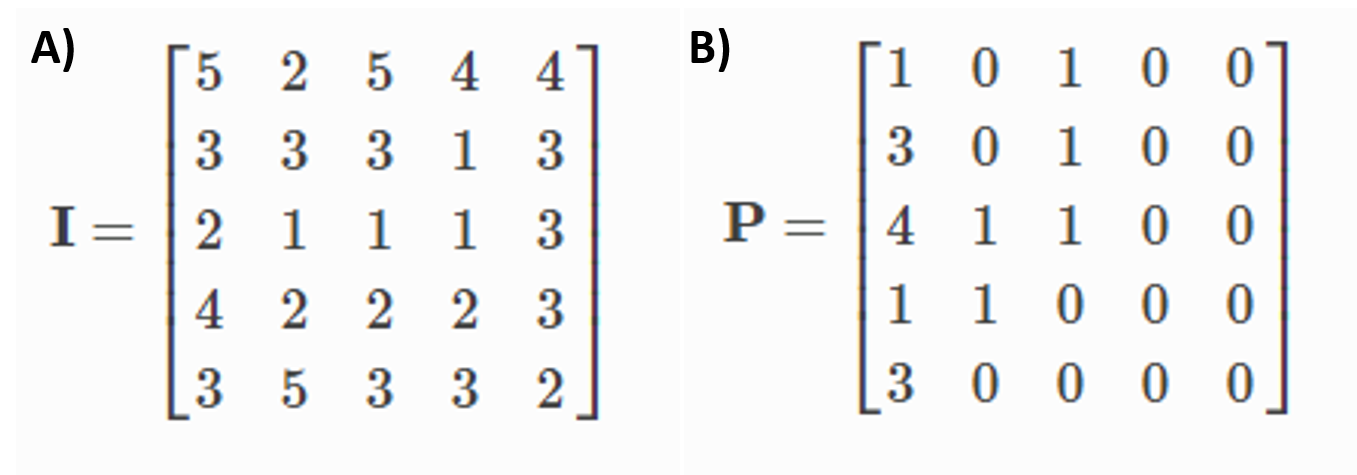
*

**Figure S2** Example of computation of a Grey Level Run Length Matrix (GLRLM). A) Image I with the original grey values. B) Corresponding GLRLM. The matrix P in B) is computed using θ = 0 (horizontal direction). Adapted from [1].

The GLSZM quantifies grey level zones in an image. A grey level zone is defined as the number of connected voxels that share the same grey level intensity. The (i; j) element of the GLSZM equals the number of zones with grey level i and size j appear in image. An example of GLSZM computation is reported in Figure S3. The GLSZM matrix displayed in Figure S3B is displayed as a 5x5 for simplicity, but in general a GLSMZ has size of N_g_-by-N_p_, where N_p_ is the total number of pixels in the image.

The NGTDM quantifies the difference between a grey value and the average grey value of its neighbors within distance δ. The NGTDM is a N_g_-by-4 matrix (Figure S4). The (i; 1) element of the matrix shows the grey value i, the elements (i; 2) and (i; 3) represent its absolute and relative frequency in the matrix and, given a size δ, the element (i; 4) is the mean absolute difference in the grey values between each voxel with intensity i and the average grey value in its neighborhood.

The GLDM quantifies grey level dependencies in an image. A grey level dependency is defined as the number of connected voxels within distance δ that are dependent on the center voxel. Given a parameter α, a voxel is said to be connected if $\left| i-j \right|\leq\alpha$. The (i; j) element of the GLDM describes the number of times a voxel with grey level i with j dependent voxels in its neighborhood appears in image. An example of GLDM is presented in Figure S5.

*
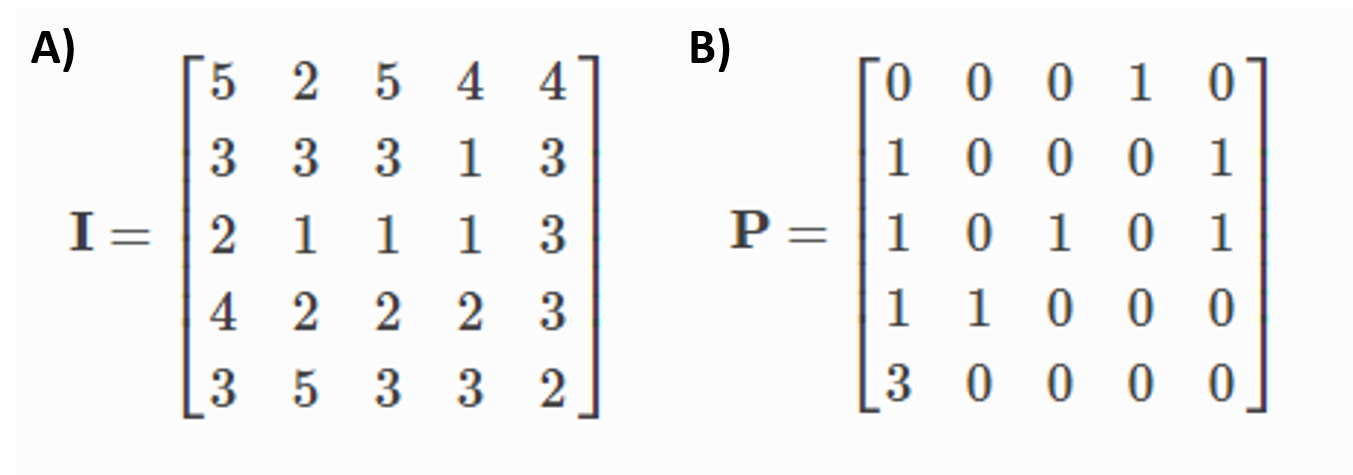
*

**Figure S3** Example of computation of a Grey Level Size Zone Matrix (GLSZM). A) Image I with the original grey values. B) Corresponding GLSZM. Adapted from [1].

*
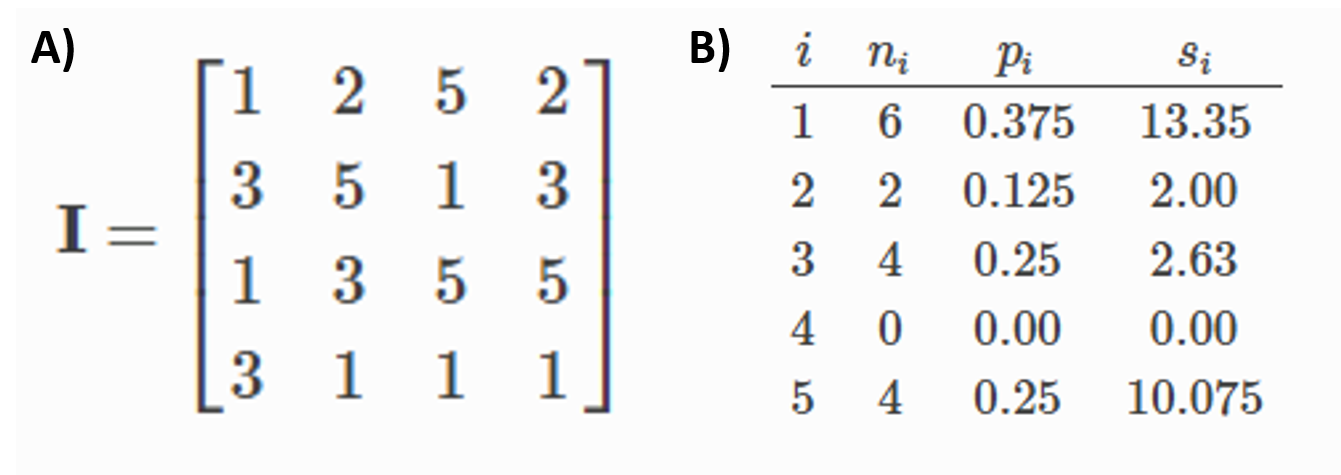
*

**Figure S4** Example of computation of a Neighbouring Grey Tone Difference Matrix (NGTDM). A) Image I with the original grey values. B) Corresponding NGTDM. Adapted from [1].

*
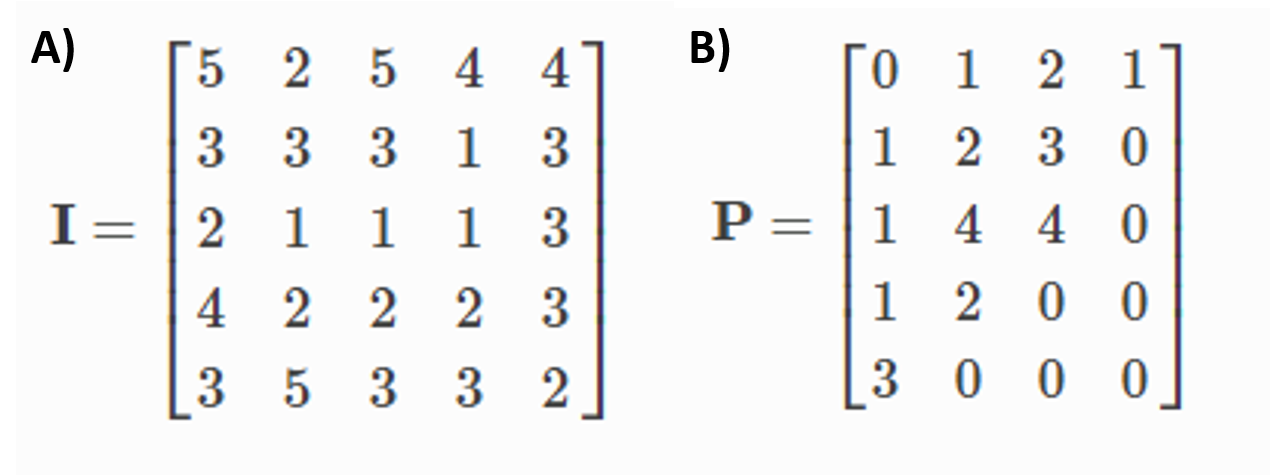
*

**Figure S5** Example of computation of a Grey Level Dependence Matrix (GLDM). A) Image I with the original grey values. B) Corresponding GLDM obtained by setting α = 0 and δ = 1. Adapted from [1].

***Wavelet transform and wavelet features***

Radiomic features belonging to FOS and textural groups may be extracted not only from the original images, but also from transformed versions, to provide additional insight on the object that is being imaged (e.g. the tumor). In this work, the wavelet decomposition was used.

Wavelet decomposition effectively decouples textural information by decomposing the original image, in a similar manner as Fourier analysis, in low and high frequencies [3]. A detailed mathematical theory of wavelet theory is beyond the scope of this material, and for that, the reader may refer to [4]. The following explanation provides the minimum knowledge to understand the applications of 2D Discrete Wavelet Transform (DWT), in image processing and in radiomics.

Essentially, the DWT of an image up to level (or scale) J is performed through a cascade tree of low-pass and high-pass filters followed by down-sampling by a factor of 2. For a 2D image, performing one level of a 2D wavelet decomposition consists of filtering and down-sampling an image I(x; y) both in the x and y directions, with both a 1D low-pass and high-pass filters. This results in four sub-bands (Figure S6): LL, LH, HL, HH. The LL band (upper-left in Figure S6) contains a coarse approximation of the original image, while the other bands contain information about high frequency changes in intensity in the horizontal, vertical and diagonal direction respectively (upper-right, lower-left and lower-right of Figure S6). Extracting the radiomic features from those sub-bands will result in new, potentially useful, information.

In case of 3D volumes, like in MRI exams, the combination of high and low-pass filters will result in 8 different possible combinations (Figure S7), and the FOS and textural features will be extracted from each of the sub-bands.


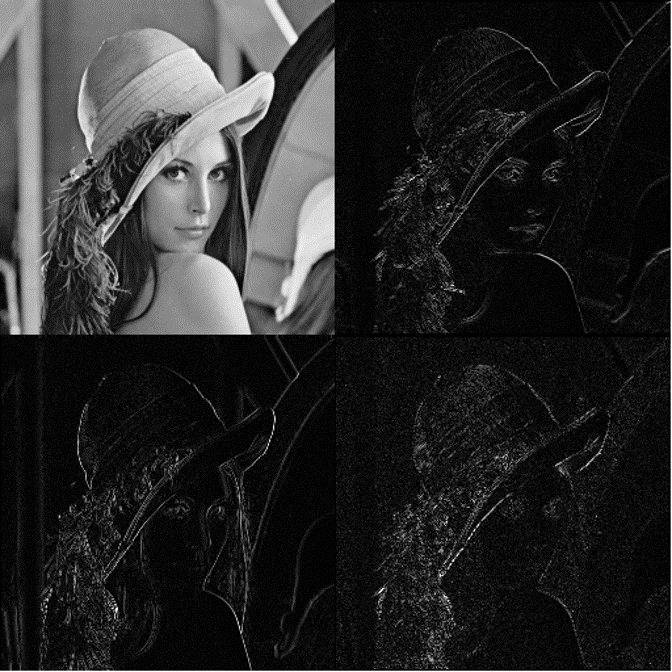


**Figure S6** Example of first level 2D discrete wavelet decomposition of an image. The upper-left corner represents the LL sub-band (an approximation of the original image). The upper-right, lower-left and lower-right parts of the image represent the LH, HL and HH sub-bands, respectively. The latter have been thresholded for better visualization.


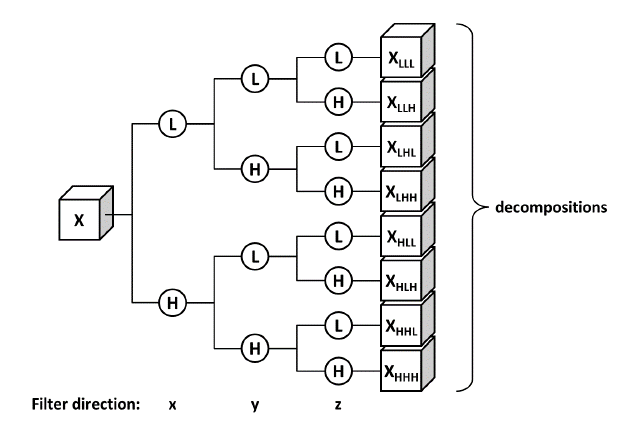


***Figure S7*** *Schematic representation of the 8 possible discrete wavelet decompositions of a 3D image [3].*

**References**

1. Pyradiomics features description, Available online: https://pyradiomics.readthedocs.io/en/2.1.0/features.html.

2. A. Zwanenburg, S. Leger, M. Vallières, S. Löck, Image biomarker standardization initiative, Reference manual available online: https://arxiv.org/abs/1612.07003.

3. H.J.W.L. Aerts, E.R. Velazquez, R.T.H. Leijenaar, C. Parmar, P. Grossmann, S. Carvalho, J. Bussink, R. Monshouwer, B. Haibe-Kains, D. Rietveld, F. Hoebers, M.M. Rietbergen, C.R. Leemans, A. Dekker, J. Quackenbush, R.J. Gillies, P. Lambin, Decoding tumour phenotype by noninvasive imaging using a quantitative radiomics approach, Nat. Commun. 5 (2014) 4006.

4. R.C. Gonzalez, R.E. Woods, Digital image processing, Prentice Hall Professional Technical Reference, 1992.
